# Perineal Perils in ED Lobby Medicine – The Importance of a Thorough Second Exam

**DOI:** 10.51894/001c.166003

**Published:** 2026-07-31

**Authors:** Mitchel Rice, Alan A. Lazzara

**Affiliations:** 1 Henry Ford - Jackson

## 117

### Introduction

Necrotizing soft tissue infections (NSTIs) are rapidly progressive life-threatening infections that require early recognition, broad-spectrum antibiotics, and prompt operative debridement to reduce mortality. Fournier’s gangrene (FG) is an NSTI sub type that classically involves the perineal, gluteal, or perigenital regions. These cases are challenging as symptoms can be nonspecific. Concealed anatomy may be difficult to visualize during limited initial examinations, which are more commonplace due to ED crowding and lobby medicine. We present a case of gluteal FG, discovered after repeat physical examination prompted by a family member’s observation.

### Case Description

A 67 yo female presented with 5 days of generalized weakness, nausea, vomiting, diarrhea, and a fall with lower back pain. She denied fevers or genitourinary symptoms. Vitals notable for tachycardia and mild hypotension. The patient was initially evaluated in a wheelchair in the ED lobby. She was non-toxic, in no distress. Limited exam was notable for mild diffuse abdominal tenderness, no abnormality to the lower back or upper buttocks. Initial work-up demonstrated WBC 32.9K, Cr 4.09, BUN 48, Lactate 2.6. While being moved to a room, a family member reported “bruising” on the buttocks not previously noted. Repeat examination revealed a 19 × 12 cm necrotic lesion extending toward the perineum. General surgery was promptly consulted, and antibiotic therapy was broadened with anaerobic and anti-toxin coverage. She was taken directly for emergent operative debridement revealing extensive gluteal and perineal necrosis with rectal perforation.

### Discussion

Our case demonstrates how an atypical presentation of NSTI in sub-optimal practice setting may lead to a delay in diagnosis. We highlight the importance of repeat evaluations in patients with infections of unclear etiology or clinical deterioration, as well as maintaining a high index of suspicion for NSTI. Once considered, NSTI’s prompt emergent surgical bedside evaluation, CT imaging, and initiation of broad spectrum antibiotics with anti-toxin coverage. A LNIREC score may be used with caution for risk stratification, although should never replace clinician gestalt or bedside surgical evaluation.

